# A Novel *HNF4A* Mutation Causing Three Phenotypic Forms of Glucose Dysregulation in a Family

**DOI:** 10.3389/fped.2020.00320

**Published:** 2020-06-26

**Authors:** Suresh Chandran, Victor Samuel Rajadurai, Wai Han Hoi, Sarah E. Flanagan, Khalid Hussain, Fabian Yap

**Affiliations:** ^1^Division of Medicine, KK Women's and Children's Hospital, Singapore, Singapore; ^2^Duke-NUS Medical School, National University of Singapore, Singapore, Singapore; ^3^Lee Kong Chian School of Medicine, Singapore, Singapore; ^4^Yong Loo Lin School of Medicine, Singapore, Singapore; ^5^Department of Endocrinology, Tan Tock Seng Hospital, Singapore, Singapore; ^6^Institute of Biomedical and Clinical Science, University of Exeter Medical School, Exeter, United Kingdom; ^7^Department of Pediatric Endocrinology, Sidra Medicine, Doha, Qatar

**Keywords:** maturity-onset diabetes mellitus, hepatocyte nuclear factor 4-alpha, hepatocyte nuclear factor−1- alpha, hyperinsulinemic hypoglycemia of infancy, congenital hyperinsulinism, diazoxide

## Abstract

Maturity-onset diabetes of the young (MODY) classically describes dominantly inherited forms of monogenic diabetes diagnosed before 25 years of age due to pancreatic β-cell dysfunction. In contrast, mutations in certain MODY genes can also present with transient or persistent hyperinsulinemic hypoglycemia in newborn infants, reflecting instead β-cell dysregulation. Of the MODY genes described to date, only hepatocyte nuclear factor-4-alpha (*HNF4A*; MODY1) and hepatocyte nuclear factor-1-alpha *(HNF1A*; MODY3) mutations may result in a biphasic phenotype of hypoglycemia in early life and hyperglycemia in later life. We report a family with a novel *HNF4A* mutation with diverse phenotypic presentations of glucose dysregulation. The proband was a term, appropriate-for-gestational age male infant with symptomatic hypoglycemia on day 3 of life needing high glucose infusion rate to maintain normoglycemia. He was born to a non-obese and non-diabetic mother. Glucose regulation was optimized using diazoxide upon confirmation of hyperinsulinism. Cascade genetic screening identified the same mutation in his father and elder sister, but mother was negative. Father was diagnosed with Type 1 diabetes at 15 years of age that required insulin therapy. Proband's elder sister, born at term appropriate for gestational age, presented with transient neonatal hypoglycemia needing parenteral glucose infusion for a week followed by spontaneous resolution. The paternal grandparents were negative for this mutation, confirming a paternal *de novo* mutation and autosomal dominant inheritance in this family. This pedigree suggests that the presence of early-onset paternal diabetes should prompt molecular testing in infants presenting in the newborn period with diazoxide-responsive hyperinsulinemic hypoglycemia, even in the absence of maternal diabetes and macrosomia.

## Introduction

Maturity-onset diabetes of the young (MODY) is an acronym used to describe dominantly inherited forms of monogenic diabetes diagnosed before 25 years of age (1). MODY gene mutations have been described with clinically heterogeneous phenotypes (2). Among these, only *HNF4A* (MODY1) and *HNF1A* (MODY3) mutations on chromosomes 12 and 20 respectively, may result in a biphasic phenotype (3). *HNF4A* is an orphan receptor protein expressed in the liver, kidney, gut, and pancreatic β-cells (4). Mutations in *HNF4A* may lead to biphasic presentations, characterized by transient or persistent HH in infants, and diabetes in young adults. *HNF4A* mutations are a less common cause of MODY (10%) than glucokinase *(GCK)* (30–50%) and *HNF1A* (30–50%). Among infants with *HNF4A* mutations, 56% are macrosomic and 15% encounter neonatal hypoglycemia (5). The prevalence of neonatal hypoglycemia is similar regardless of parental *HNF4A* inheritance and persistent hypoglycemia is independent of gestational glucose control (6). Hyperinsulinemic hypoglycemia (HH) is characterized by inappropriate insulin secretion while hypoglycemic and the need for high glucose infusion rate (GIR) requirements (>8 mg/kg/min) to maintain normoglycemia (3.5–5.9 mmol/L) in newborn infants beyond 48 h of life. HH infants have inappropriate insulin and/or C-peptide levels despite the presence of hypoglycemia, hypoketonemia and hypofattyacidemia. HH is linked to mutations in at least eight genes (*ABCC8, KCNJ11, GLUD1, GCK, HADH, SLC16A1, HNF4A, HNF1A*) that alter β-cell function (7). Unlike the majority of mutations involving the K_ATP_ channel, *HNF4A* mutations causing HH respond well to diazoxide (8). We present a family with a novel *HNF4A* mutation identified from a proband presenting with symptomatic hypoglycemia. The family members were found to have contrasting phenotypic presentations of glucose dysregulation in spite of having the same mutation. We present this pedigree to demonstrate that a history of paternal diabetes is as important as a history of maternal diabetes, and relevant in pediatric history-taking when managing infants at-risk of hypoglycemia.

## Case Presentation

### Pedigree Report

A healthy male infant weighing 3,592 gm was born to non-consanguineous parents at 37+5 weeks gestation and discharged uneventfully on day 2 of life. Both parents are of Malay ethnicity. Maternal health during pregnancy and oral glucose tolerance test results were normal. There was no maternal family history of diabetes. On day 3 of life, this infant was admitted for treatment of neonatal jaundice. While on phototherapy he was noted to be apneic and cyanosed with low plasma glucose (1.8 mmol/L). Resuscitation involved mini bolus intravenous 10% dextrose and continuous glucose infusion. As the response was inadequate, GIR was graded up to 16 mg/kg/min before glucose levels normalized.

Physical examination of the infant was unremarkable. Upon reduction of GIR in a controlled setting, critical blood tests were obtained that showed detectable C- peptide [1.7mcg/L] and insulin [10.4 mU/L] when plasma glucose was 1.6 mmol/L. Along with suppressed blood ketones of 0.2 mmol/L, these indicate inappropriate insulin production during hypoglycaemia, which fulfilled the diagnostic criteria for HH. Serum cortisol was 494 nmol/L and GH level was 13.4 ug/L, showing adequate pituitary response. Septic and inborn error of metabolism screens were negative. On day 7 of life, he was started on diazoxide, 5 mg/kg/day in divided doses, with normoglycemia achieved after titrating diazoxide up to 10 mg/kg/day on day 19 of life. Hydrochlorothiazide was added to counteract the salt and water retaining side effects of diazoxide. Thereafter, his GIR was weaned over 5 days and he remained normoglycemic on full oral feeds. Before discharge while on diazoxide, he passed a 6-h safety fast study, to reassure of his ability to maintain glucose levels during inadvertent fasting periods at home. Glucose monitoring continued at home. The absolute diazoxide dose was maintained, allowing weight-based reduction of the dose toward 24 months of age. At 36 months, diazoxide was stopped and he underwent and passed a resolution fasting study. Growth and neurocognitive development are currently appropriate for age.

**Figure 1:**
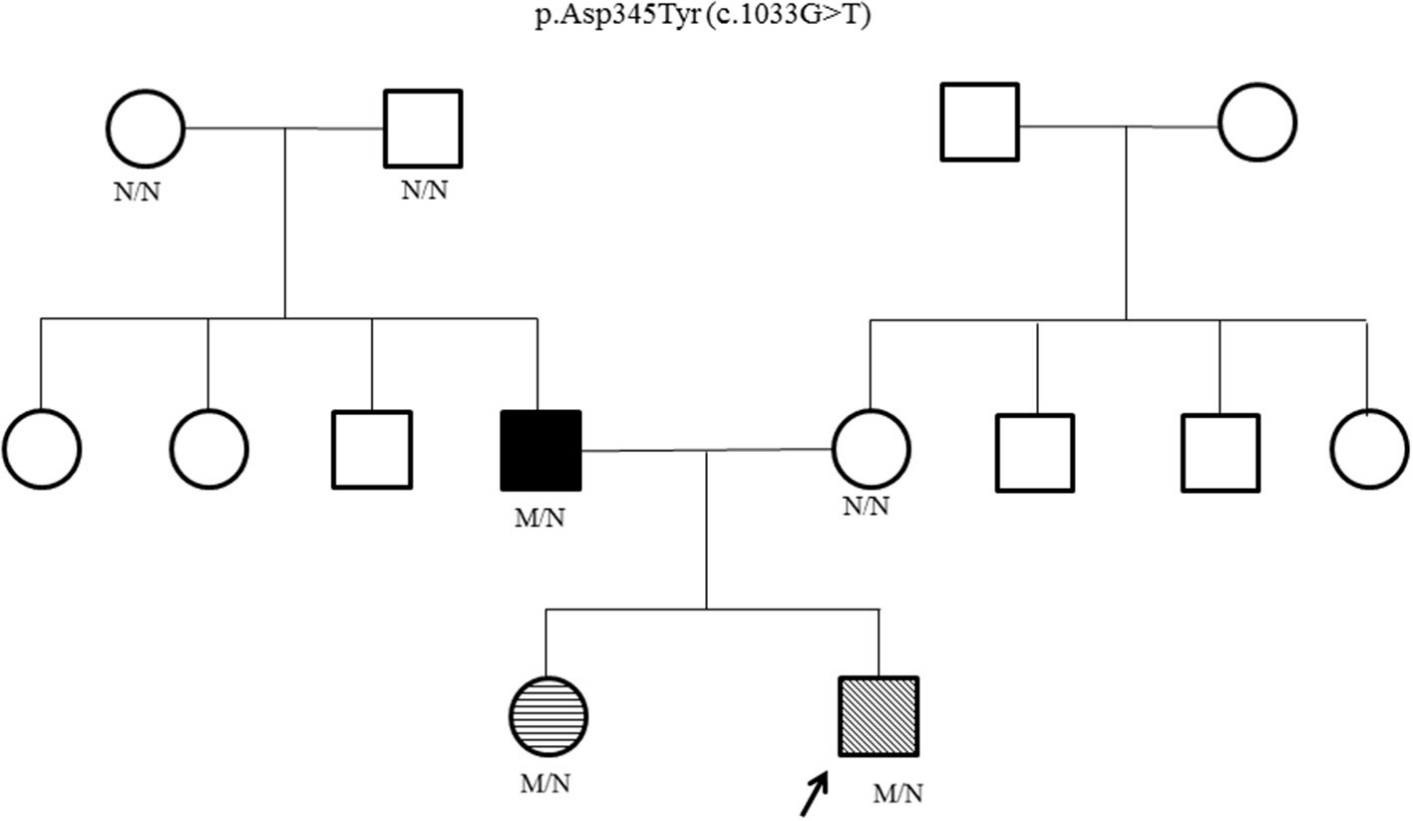
*HNF4A* family pedigree. Circles (females) and squares (males). Arrow marks the Proband. Filled symbol indicates MODY (father); unfilled symbol indicates no history of glucose dysregulation. Diagonal hatching denotes persistent HH (proband). Transverse hatching indicates transient hypoglycemia (sister). M/N = heterozygous *HNF4A* genotype; N/N = normal genotype.

Genetic testing for hyperinsulinism was performed at Exeter, UK. Analysis of coding and flanking intronic regions of the *KCNJ11, ABCC8*, and *HNF4A* genes by Sanger sequencing was done. Both *KCNJ11* and *ABCC8* genes were normal. A novel heterozygous *HNF4A* missense mutation, p.Asp345Tyr (c.1033G>T) was identified. Cascade family screening identified the same *HNF4A* mutation in his father and elder sister; whereas his mother and paternal grandparents were negative (Figure 1). This confirmed that the proband's father has a *de novo* mutation, consistent with MODY rather than type 1 diabetes.

**Figure 2:**
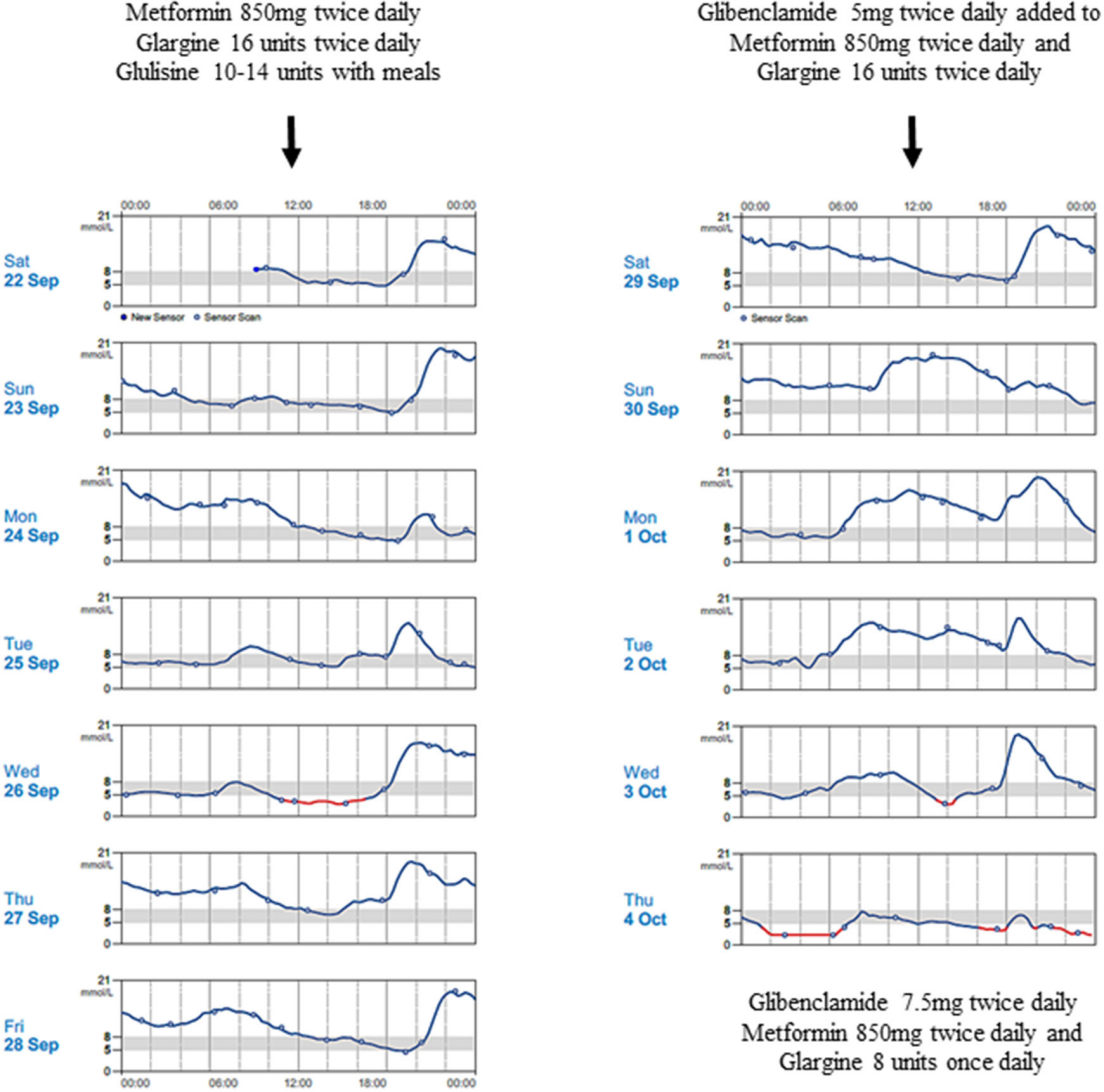
Continuous blood glucose patterns of the proband's father, capturing his daily glucose variability over 2 weeks, while he was on Metformin 850 mg twice daily, Glargine 16 units twice daily and Glulisine 10–14 units thrice daily (first week); and demonstrating improved fasting glucose levels despite progressive reduction in Glargine doses after Glibenclamide was progressively introduced (second week).

The proband's father was diagnosed with diabetes at age 15 years and treated with insulin. He was obese from early childhood, even though there was no history of diabetes in his parents or siblings. Details of initial management prior to his transfer to tertiary diabetes care are unavailable. At age 33 years, his glycated hemoglobin (HbA1c) was 12%, fasting glucose 18.3 mmol/L, C-peptide 270 pmol/L (364-1655), glutamic acid decarboxylase (GAD) autoantibody and islet-cell autoantibody tested negative. Diabetes control was suboptimal due to poor adherence. On Metformin 850 mg twice daiy (BD) and basal-bolus insulin therapy, HbA1c ranged from 6.9 to 10% over the next 5 years. At age 38 years, he was reassessed following his son's genetic diagnosis. While on Metformin 850 mg BD, subcutaneous (SC) Glargine 16u BD and SC Glulisine 10–14u thrice daily (TDS), HbA1c was 9%, fasting glucose 16.5 mmol/L and C-peptide 481 pmol/L. He measured 1.54 m, weighed 86 kg, giving a BMI of 36.3 kg/m^2^. Given his reasonable insulin reserve, sulphonylurea therapy was initiated to determine if insulin doses could be reduced without compromising glucose control. Ambulatory glucose profiles were conducted over a 2-week period—in the first week, he was on his usual treatment regimen while in second week, sulphonylurea was added (Figure 2). He responded to up-titrated doses of Glibenclamide with reduction of basal insulin from 32 to 8 units daily, while maintaining similar glucose profiles. Over these 2 weeks, the composite ambulatory glucose profile showed average glucose of 9.6 mmol/L, giving an estimated HbA1c of 7.7%. His current medication doses are Glibenclamide 7.5 mg BD, Metformin 850 mg BD, SC Glargine 8 units every night (ON) and SC Glulisine 10u BD. After glibenclamide was added and the basal insulin dose was reduced, there was improvement in his fasting glucose levels from 11-13 mmol/L to 5-6.4 mmol/L. Despite subsequent increments in sulphonylureas, he required prandial (albeit decreased) doses of glulisine for persistent postprandial hyperglycemia. His total daily insulin requirements decreased from 1 to 0.5 u/kg/d. The incomplete response to sulphonylureas was likely due to progressive defect in beta cell β-cell dysfunction after having had diabetes for 23 years. His most recent BMI was 36.4 kg/m^2^.

**Figure 3:**
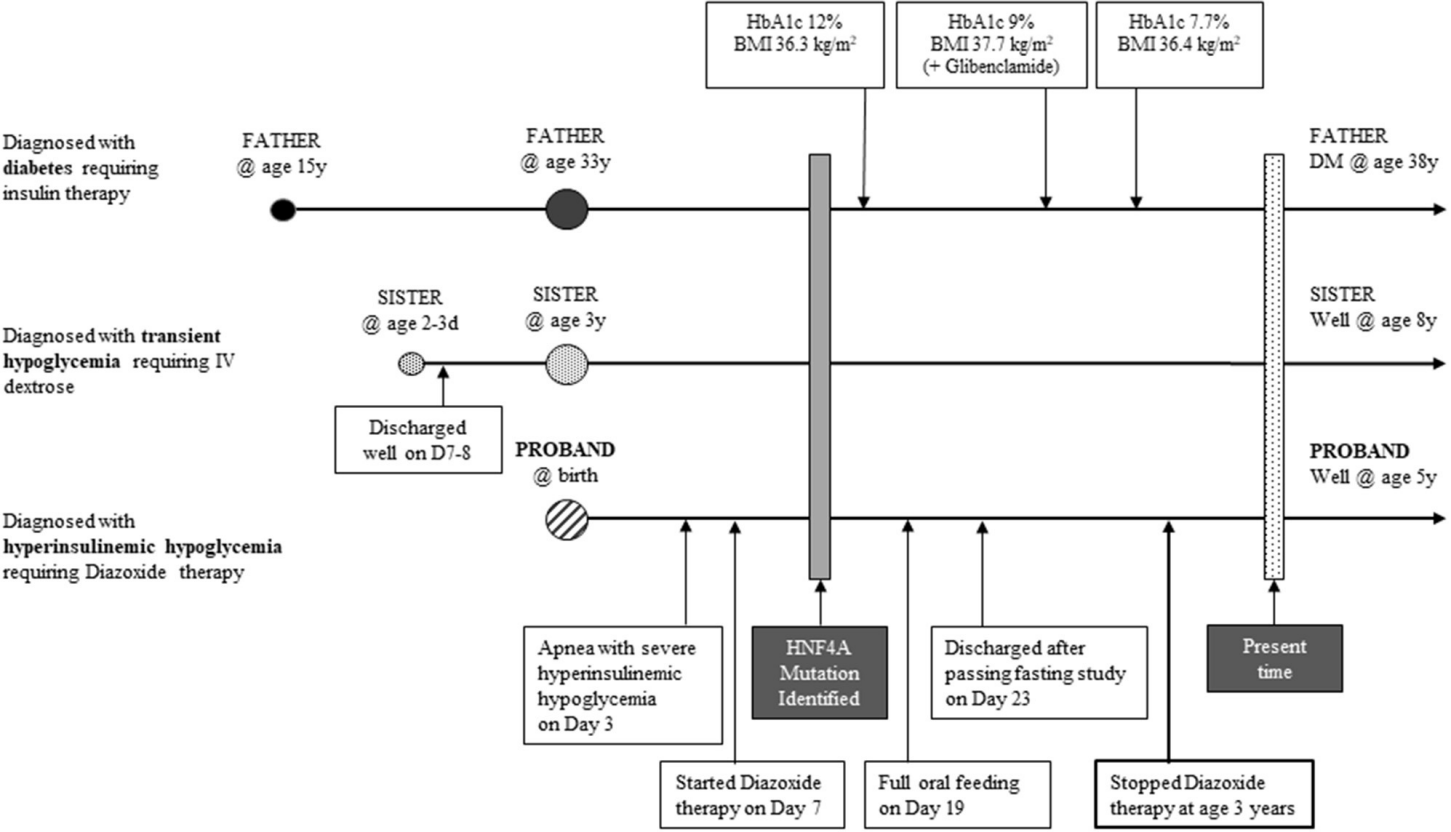
Timeline depicting the three phenotypes of this pedigree.

The proband's 8 year old sister who was born term, appropriate for gestational age (birth weight, 2835 g) had transient hypoglycemia during neonatal period. Work up for sepsis and inborn error of metabolism screen were negative. However, her phenotype was mild and she required intravenous dextrose (highest GIR 7.6 mg/kg/min on day 4 of life), before gradual increase in feeds normalized her blood glucose levels by day 7 of life. She has appropriate growth and remains in a mainstream school (Figure 3).

### Family's Perspective

Upon receiving news of the genetic diagnosis, the proband's father was hopeful for better control with reduced insulin. Although his endocrinologist was able to use this information to adjust his treatment regimen, he experienced difficulty making lifestyle changes which limited his glucose control and prevented further reduction of his medications. The proband's mother was more concerned about the implications of the diagnosis on her children. She was particularly happy that the proband did not require long-term Diazoxide therapy, although she was concerned of the risk of diabetes in both her children. The proband's paternal grandparents expressed that the exact diagnosis makes them aware of the risk of childhood-onset diabetes. They clearly indicated their hope for their grandchildren to remain healthy and not to develop obesity and childhood-onset diabetes like their son (the proband's father) did.

## Discussion

We describe a pedigree where a heterozygous novel *HNF4A* mutation was identified in 3 individuals of a 2 generation family. Each of these individuals were phenotypically distinct—the proband had persistent HH, his sister had transient hypoglycemia, while his father had juvenile-onset MODY. The paternal grandparents tested negative, confirming a spontaneous *de novo* mutation, followed by dominant inheritance. Identification of the underlying genetic etiology allowed for a molecular diagnosis of the proband, clarified the paternal phenotype as MODY instead of type 1 diabetes and facilitated his improved diabetes management.

Up to 80% cases of diabetes due to MODY gene mutations are misclassified as type 1 or type 2 diabetes, leading to inappropriate medical therapy (9). The diagnosis of MODY requires molecular confirmation (10). In retrospect, precise molecular diagnosis of the diabetes in the proband's father would have permitted close fetal monitoring for macrosomia and appropriate postnatal glucose surveillance. Mutations in *HNF4A* are reported to be highly penetrant with 50% of carriers developing diabetes by age 30 years, whereas 60% with *HNF1A* mutations present by 25 years (11). The father of the proband was diagnosed with diabetes at age 15 years, expressing the highly penetrant nature of this novel *HNF4A* mutation. Infants who inherit *HNF4A* have significantly increased birth weight, with more than half having macrosomia. The risk of macrosomia is higher in maternally-inherited mutations (64%) compared to paternal inheritance (46%), due to the additional effect of hyperglycemic intrauterine milieu (6). The paternal inheritance pattern in this pedigree may explain the absence of macrosomia in both his offsprings.

HNF4A mutations are the third most common genetic cause of diazoxide-responsive HH, (8) however the mechanism by which these mutations cause insulin excess in fetal and neonatal life and insulin deficiency later in life is unclear. Heterozygous loss-of-function mutations in *HNF4A* may cause either transient or persistent HH (6, 12). Current evidence supports a reduction in expression of inward rectifying potassium channel subunit (Kir 6.2) and/or reduction in the levels of peroxisome proliferator-activated receptor alpha (PPARα), causing inappropriate insulin secretion and resulting in HH in newborn period (13, 14). Low levels of PPARα have been reported in *HNF4A* deficient β-cells, resulting in the accumulation of lipids and thereby increasing cytosolic long-chain acyl-CoA levels, signaling insulin release. Long-term exposure of β-cells to elevated concentrations of fatty acids causes β-cell dysfunction leading to diabetes (15). Other suggested theories for the dual phenotype in *HNF4A* mutations include variance in *HNF4A* dependent temporal gene expression, β-cell exhaustion from hypersecretion in fetal life and infancy and malfunction of transcription factors that sustain β-cell function in pancreatic islets (7, 16, 17). MODY responds well to low-dose sulfonylurea, maintaining the glucose profile even after 3 decades (16, 18). In individuals with a *HNF4A* mutation, as the β-cell dysfunction is progressive with age, insulin treatment may eventually be required. If the proband's father had received an early genetic diagnosis, he may have benefitted from oral sulfonylurea instead of insulin. Secondary sulphonylurea failure has been described to occur in 3 to 25 years following diagnosis/treatment in transcription factor linked-MODY patients (19). Unlike *GCK* mutations, patients with diabetes due to *HNF4A* and *HNF1A* mutations are at increased risk of micro and macrovascular complications, (6, 9, 18, 20) and therefore require early and sustained glucose control.

Diazoxide remains the first line of medical treatment for HH. Diazoxide response in HH due to a *HNF4A* mutation is adequate but the treatment period may vary from months to years (9). In this proband with a novel *HNF4A* mutation, glucose levels were controlled with moderate doses of diazoxide weaned over 3 years. Following cessation of therapy, resolution of HH was confirmed with a fasting study. As there is potential of developing MODY, glucose tolerance testing is planned for the proband and his sister.

The strength of this case study lies in the full phenotypic characterization of the proband and cascade genetic testing in his family. However, type 1 diabetes management details of the father are unavailable prior to the proband's diagnosis. The proband's sister was not evaluated for hyperinsulinism as she did not meet the criteria for HH.

In conclusion, we present a family with a novel *HNF4A* mutation having 3 phenotypic presentations across 2 generations. This case pedigree supports *HNF4A* gene sequencing for infants presenting in the newborn period with diazoxide-responsive HH and paternal diabetes, even in the absence of maternal pre or gestational diabetes and fetal macrosomia. Those with prior molecular diagnosis of *HNF4A* mutation should be monitored for early diagnosis of sulfonylurea-sensitive diabetes, to allow earlier intervention and treatment. Overall, an early molecular diagnosis of *HNF4A* MODY can guide a change in therapy from insulin to sulfonylurea, improving the lifestyle and quality of life for both patients and their families.

## Data Availability Statement

The datasets generated for this study can be found in the HNF4A mutation details have been deposited in the Decipher database (https://decipher.sanger.ac.uk/). The raw dataset can however be used to identify individuals and so cannot be made openly available. Access to data is open through collaboration. Requests for collaboration will be considered following an application to the Genetic Beta Cell Research Bank (https://imsva91-ctp.trendmicro.com:443/wis/clicktime/v1/query?url=https%3a%2f%2fwww.diabetesgenes.org%2fcurrentresearch%2fgenetic%2dbeta%2dcell%2dresearch%2dbank%2f&amp;umid=1C0AE20F-9E54-0305-9291-12237D74D356&amp;auth=6e3fe59570831a389716849e93b5d483c90c3fe4-5a72b0906f4668ba59b7efa43cf143736ad6be6c). Contact by email should be directed to the lead nurse, Dr. Bridget Knight (b.a.knight@exeter.ac.uk).

## Ethics Statement

Ethical review and approval was not required for the study on human participants in accordance with the local legislation and institutional requirements. Written informed consent to participate in this study was provided by the participants' legal guardian/next of kin. Written informed consent was obtained from the individual(s), and minor(s)' legal guardian/next of kin, for the publication of any potentially identifiable images or data included in this article.

## Author Contributions

SC and FY treated the infant, wrote the manuscript, and performed the final edits. WH treated the father of the index case and wrote the paternal case section. VR reviewed and revised the manuscript critically for important intellectual content. SF and KH performed the genetic testing for the pedigree, contributed to the genetic section of the manuscript and reviewed the manuscript. All authors approved the final manuscript as submitted and agree to be accountable for all aspects of the work.

## Conflict of Interest

The authors declare that the research was conducted in the absence of any commercial or financial relationships that could be construed as a potential conflict of interest.
